# Rel-A/PACER/miR7 Axis May Play a Role in Radiotherapy Treatment in Breast Cancer Patients

**DOI:** 10.61186/ibj.3901

**Published:** 2023-05-08

**Authors:** Fazlollah ShokriShokri, Hossein Mozdarani, Mir Davood Omrani

**Affiliations:** 1 *Department of Medical Genetics, Faculty of Medical Sciences, Tarbiat Modares University, Tehran, Iran;*; 2 *Urogenital Stem Cell Research Center, Shahid Beheshti University of Medical Sciences, Tehran, Iran*

**Keywords:** Breast cancer, Radiation biomarkers, Radiotherapy

## Abstract

**Background:**

Radiotherapy has become the standard form of treatment for BC. Radioresistance is an issue that limits the effectiveness of RT. Therefore, predictive biomarkers are needed to choose the appropriate RT for the patient. Activation of the proinflammatory transcription factor, *NF-κB*, is a frequently noted pathway in BC. Investigating the relationship between RT and alterations in gene expression involved in the immune pathway can help better control the disease. This research investigated the impact of RT on the expression levels of *Rel-A*,* PACER,* and *miR-7 *within the *NF-κB* signaling pathway.

**Methods:**

Blood samples (n = 15) were obtained from BC patients during four different time intervals: 72 hours prior to initiating RT, as well as one, two, and four weeks following RT completion. Samples were also collected from 20 healthy women who had no immune or cancer-related diseases. Blood RNA was extracted, and cDNA was synthesized. Gene expression level was determined using RT-PCR.

**Results:**

There was a significant difference in the expression level of *Rel-A* between patients and normal individual blood samples (p < 0.05). After four weeks of RT, qRT-PCR revealed a significant downregulation of *miR-7* and upregulation of *Rel-A* and *PACER* in BC patients. Also, there was a significant association between *Rel-A* expression and monocyte numbers during RT (p < 0.001).

**Conclusions:**

The expression level of* PACER*, *miR-7* and *Rel-A*, changed after RT; therefore, these genes could be used as diagnostic and therapeutic RT markers in BC.

## INTRODUCTION

Tumor-promoting inflammation is one of the hallmarks of cancer. It has long been recognized that there is a strong relationship between the presence of inflammation, *NF-κB* signaling, and the development of premalignant lesions in varied sites^[^^1^^-^^3^^]^. 

The *NF-κB/REL* family of transcription factors is composed of *Rel-A/p65*, *c-REL*,* RELB*,* p105/NF*, and *p100/NF-κB2*^[^^4^^]^. Members of this family are distinguished by the presence of the *REL* homology domain (responsible for the binding and translocation of the sequence-specific DNA) in the N-terminus. The C-terminal regions of these proteins consist of domains that are involved in either transcriptional activation (*Rel-A*,* c-REL*, and *RELB*) or inhibiting *Rel *proteins (*p105 *and* p100*). The *REL* family members form a variety of homodimers and heterodimers, among them, the most common one is the *p65/p50*, also known as the *NF-κB* complex^[^^5^^]^. Activation of the *NF-κB* signaling pathway leads to the promotion of specific genes, which have the ability to prevent apoptosis, influence the regulation of the cell cycle, contribute to the development of tumors and inflammation and promote metastatic growth^[^^6^^]^. *NF-κB* activation in BC causes the loss of ER expression and overexpression of human epidermal growth factor receptor-2 via the *EGFR* and mitogen-activated protein kinases pathway^[^^7^^,^^8^^]^. *NF-κB* is also known to reduce the cell death in response to radiation by promoting the expression of antiapoptotic proteins and activating the cellular antioxidant defense system. It is well known that constitutive activation of *NF-κB*-associated genes in tumor cells enhances radiation resistance. Following radiation exposure, *NF-κB* regulates the production of a wide range of cytokines and chemokines, which assists in enhancing cell proliferation and tissue regeneration in various organs^[^^9^^]^. Since *NF-κB*-regulated genes play roles in invasiveness, proliferation, angiogenesis, lymphatic angiogenesis, and inflammation, they present themselves as promising candidates for elucidating different aspects of BC progression^[^^10^^,^^11^^]^. Increasing evidence has suggested that *NF-κB*-associated pathways are dysregulated in numerous malignancies, including BC^[^^12^^,^^13^^]^. However, the underlying mechanisms for abnormal *NF-κB* activation in BC are not completely understood. There is growing evidence that lncRNAs such as *PACER* is involved in the dysregulation of NF-κB signaling^[^^14^^]^. *PACER* interacts directly with *p50*, which can form active heterodimers with *p65/RelA* and also inactive *p50/p50* homodimers that lack the transcription activation domains found in *p65/RelA* during the normal course of *NF-κB* pathway activation^[^^14^^,^^15^^]^.

Small non-protein-coding RNAs are considered miRNAs bind to the 3'-UTRs of target mRNAs to repress the translation of the target genes. The human genome contains cancer-related regions where miRNA genes can be found. Through the endogenous RNA interference pathway, a large number of miRNAs regulate the expression of oncogenes or tumor suppressor genes^[^^16^^]^. In contrast, *hsa-miR-7-5p* has been demonstrated to act as an inhibitor of BC invasion and metastasis and facilitate the destruction of BC cells through cytotoxic T-lymphocyte. It has also been displayed that *miR-7* causes apoptosis and inhibits cell proliferation in various BC cell lines. According to studies, *miR-7* is a tumor suppressor gene and its expression significantly decreases the capacity of BC stem cells to self-renew^[^^17^^-^^19^^]^. In cancer tissues, compared to the surrounding noncancerous tissues, *ESAM* expression is markedly upregulated; however, *miR-7* expression is considerably downregulated. *Rel-A* expression is positively correlated with *ESAM* expression in BC tissues. Furthermore, an inverse relationship has been found between the expression levels of *miR-7* and *ESAM*^[^^20^^]^. *miR-7* had a direct binding site at the 3'-UTR of *Rel-A*. The *miR-7* directly suppresses the expression of *Rel-A*, which subsequently indirectly inhibits the expression of *ESAM*. Moreover, it has been suggested that *Rel-A* expression is noticeably higher in the BC tissues than the surrounding healthy tissues^[^^20^^]^. Increasing the expression level of *miR-7* makes cancer cells more sensitive to radiation. In cancers where this signaling network is active, therapeutic upregulation of *miR-7* expression may be an effective strategy for overcoming therapeutic radioresistance^[^^16^^]^.

In the present study, our aim was to investigate how radiotherapy impacts the *NF-κB* signaling pathway among the BC patients who received RT.

## MATERIALS AND METHODS

**Bioinformatics study**

**Fig. 1:**
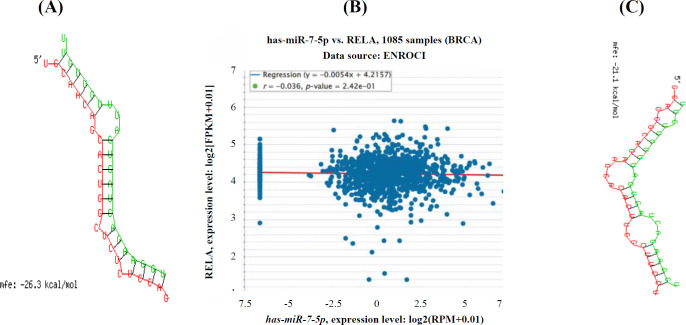
The interaction between *miR-7-5p* and its target gene *Rel-A*. Panel (A) indicates evidence of this interaction, while panel (B) represents the sequences of *Rel-A* that are targeted by *miR-7-5p*. Panel (C) displays evidence of *miR-7-5p* acting as a sponge with *PACER*.

To find the target genes that *miR-7-5p* may regulate them, we performed a bioinformatics analysis. The increased miRNA expression enhances the radiation sensitivity of cancer cells. The TCGA database was used to assess the expression of miENAs in BC patients. The miRNA targets, as shown in Figure 1, were predicted using miRTarBase (https://mirtarbase. cuhk.edu.cn/). 

**Collection of blood samples**

Only BC patients with no previous chemotherapy or RT were enrolled in the study. A total of 15 BC patients who were referred to Imam Khomeini Hospital in Tehran, Iran, between 2020 and 2021. Sample of peripheral blood (5 ml) was collected prior to the administration of RT. Blood samples were collected from the same patients during the first, second, and fourth weeks of the RT treatment. All the patients were undergone RT with a daily dose of 1.8 Gy (dose per fraction: 1.8 Gy) using an Elekta Precise linear accelerator (Varian, Sweden). The radiation doses in the treatment phases I, II, and III were 9, 9, and 18 Gy (36 Gy in the fourth week) for all the patients (Table 1). RT was performed as a three dimensional-conformal protocol with 18-MV photon beams five days per week. Furthermore, no specific medication was prescribed for the patients during RT. Blood samples were also collected from 20 healthy female volunteers with no history of malignant or inflammatory conditions in an outpatient facility. 

**Table 1:** Details of radiotherapy doses used in this study

**Phases ** **of treatment**	**Number of ** **sessions**	**Dose per fraction (Gy)**	**Dose in per phase (Gy)**
I	5	1.8	5 × 1.8 = 9
II	5	1.8	5 × 1.8 = 9
III	10	1.8	10 × 1.8 =18
Total	20	1.8	20 × 1.8 = 36

RNA extraction

Total RNA was extracted from the whole blood using EZ-RNA Reagent (BioBasic, Canada) according to the manufacturer’s protocol. RNA was diluted by 30 μl of RNase-free water and stored at -80 °C. 

Evaluating the quality and quantity of RNA 

The 260/280 and 260/230 ratios of absorbance values were used to assess the purity of RNA using a Nanodrop (Thermo Fisher Scientific, USA). A 260/280 ratio of ~2.0 and a 260/230 ratio of 2.0-2.2 were accepted as "pure" for RNA. 

**Reverse transcription and qRT-PCR**

cDNAs were synthesized using Moloney murine leukemia virus enzyme (Sinaclone, Iran), random hexamers, and stem-loop primers according to the manufacturer’s instructions. qRT-PCR was performed by specific primers (Table 2) using the StepOne Real-Time PCR system (Applied Biosystems, USA). B2M, small nucleolar RNA and C/D box 48 were used as internal controls for the normalization of mRNAs and miRNA expression. The relative quantification method (2^-ΔΔCT^) was used to calculate the fold changes of cDNA. A PCR assay was carried out duplicated and repeated two times.

**Evaluation of the number of immune cells**

Complete blood counts with differential measurements were accomplished in normal individuals and BC patients before and after RT. The clinical laboratory tests (complete blood count) were performed for all the participants at the Department of Laboratory Medicine at Imam Khomeini Hospital. The entire samples were hand-delivered within four hours of the sampling.

**Statistical analysis**

 The results were reported in the form of descriptive statistics for indicators, as number (percentage) for the qualitative variables, as well as means ± SD for the quantitative variables. To compare the expression of genes between the patients and healthy individuals, an independent t-test and analysis of variance of the repeated measures (repeated measure ANOVA) were used, respectively. For data analysis, SPSS version 19 software was employed. A significant level was defined as the probability value of less than 5% (p < 0.05). 

## RESULTS

Of 21 women screened for this study, 16 met our diagnostic and temporal inclusion criteria. Among these 16 study subjects, 15 completed the pre-RT baseline and the three-week post-RT observational study and were included in the analysis. Their age ranged from 32 to 61 years. The average age of the patients was 40.48 ± 7.3, while that of the controls was 42.21 ± 6.2. Most of the patients had estrogen (86.7%) or progesterone (80%) receptor-positive breast tumors, 86.7% had invasive ductal carcinoma, and 13.3% had both ductal carcinoma in situ and invasive lobular carcinoma. *HER2/neu* overexpression was present in 33.3% of the participants’ biopsied tissue samples. Patients’ clinicopathological details are shown in Table 3.

**Table 2:** Primer sequences used in this study

**Gene**	**Primer sequences**
*Rel-A*	F: 5′-CCAGACCAACAACAACCCCT
	R: 5′-TCACTCGGCAGATCTTGAGC
	
*PACER*	F: 5′-TGTAAATAGTTAATGTGAGCTCCA
	R: 5′-GCAAATTCTGGCCATCGC
	
*B2M*	F: 5′- CCACTGAAAAAGATGAGTATGCCT
	R: 5′- CCAATCCAAATGCGGCATCTTCA
	
*miR-7*5p	F: 5′-GGCGGTGGAAGACTAGTGATT
	STL: 5′-GTCGTATCCAGTGCAGGGTCCGAGGTATTCGCACTGGATACGACACAACAA
	
*U48*	F: 5′- GAGTGATGATGACCCCAGGTAA
	STL: 5′- GTTGGCTCTGGTGCAGGGTCCGAGGTATTCGCACCAGAGCCAACGGTCAG

**Analysis of gene expression**

Peripheral blood was collected from each of the 15 RT-treated BC patients before, and one, three, and four weeks after the initiation of therapy.

***Rel-A*** **,** ***PACER*** **, and** ***miR7*** **expression in BC patients and controls**

**Fig. 2:**
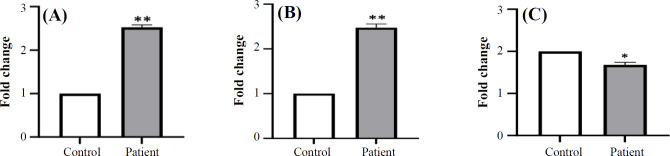
Expression of *Rel-A* (A), *PACER* (B), and *miR7* (C) in BC patients and healthy individuals.

Gene expression analysis was performed on the corresponding blood samples from BC patients and healthy individuals. The results were normalized to the endogenous control genes and expressed in relative quantity. Expression of *Rel-A* and *PACER* significantly elevated in BC patients compared to the healthy individuals (p < 0.05, Fig. 2A and 2B); however, *miR-7* expression significantly decreased in the patients’ blood samples compared to the normal samples (Fig. 2C).

***Rel-A*** **,** ***PACER*** **, and** ***miR7*** **expression in samples from RT-treated BC patients**

**Fig. 3:**
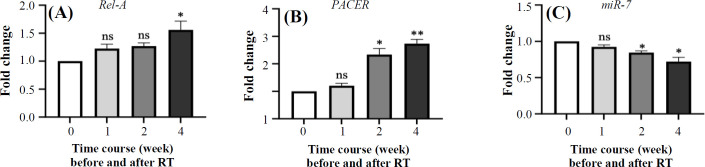
The gene expression of *Rel-A*, *PACER*, and *miR-* *7* measured before and after radiotherapy at different time points. In the first week, there were no significant changes in *Rel-A* and *miR-7* level compared to that before treatment. However, in the second and  fourth weeks, with radiation doses of 18 Gy and 36 Gy respectively, significant changes in the gene expression level were observed  (A, C). For *PACER*, no significant changes were observed in the expression levels during the first and second weeks of radiotherapy; however, after receiving 36 Gy of radiation, a significant increase in *PACER* expression was found in the fourth week (B). ns, not significant

The *Rel-A* expression level showed a significant increase in the fourth weeks following the initiation of RT. while expression level of *miR-7*, had a decreasing trend, which was insignificant for the first week. It is noteworthy that the expression level of *miR-7* decreased significantly in the second and fourth weeks after RT. No significant difference was found in the expression level of *PACER* before RT and in the first and second weeks after RT. Although its expression increased significantly in the fourth week after RT (Fig. 3).

**Table 3:** Characteristics of patients’ tumor sample

**Characteristic**	**Detail**	**Frequency**	**Percentage**
Type of breast	Left/right	1	6.7
	Left	5	33.33
	Right	9	60.0
			
ER	-	2	13.3
	+	13	86.7
			
PR	-	3	20.0
	+	12	80.0
			
HER2	-	10	66.7
	+	5	33.3
			
Grade	1-3	1	6.7
	2	6	40.0
	2-3	3	20.0
	3	5	33.3
			
Size	<=2 cm	4	26.7
	2-4 cm	7	46.7
	>4 cm	4	26.7
			
Stage	IA	2	13.3
	IIA	4	26.7
	IIB	3	20.0
	IIIA	5	33.3
	IIIC	1	6.7
			
Type	DCIS	1	6.7
	IDC	13	86.7
	ILC	1	6.7

**Association between the** **expression level of** ***Rel-A*** **and** ***PACER*** **and poor prognosis in BC** **patients**

**Fig. 4:**
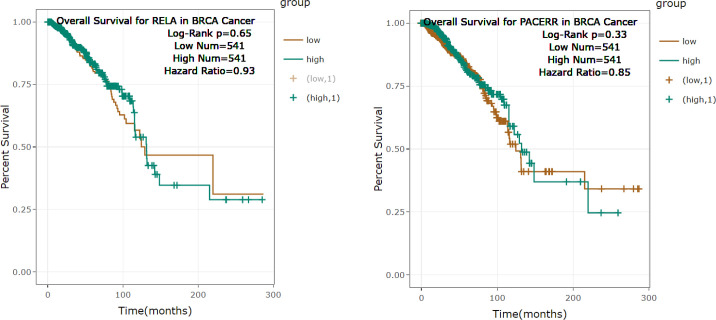
Overall survival reductions for BC patients expressing *Rel-A* and *PACER*. Patients with higher expression level of both *Rel-A* and *PACER* showed a decreased survival rate.

To explore the connection between *Rel-A* and *PACER* expressions and the survival rate of patients with BC, we selected the TCGA database. The data indicated a link between the high-level expression of *Rel-A* and *PACER* with the low overall survival rate (Fig. 4). 

**Changes in white blood cells associated with RT**

The independent t-test showed that there was a significant difference in the average neutrophil levels between the patient and control groups during the fourth week of RT (p = 0.003). In addition, the mean monocyte level was significantly different between the two groups during treatment (p = 0.042). Specifically, the average level of monocytes in the patient group was higher than that of the healthy group (p < 0.05). Additionally, in the group of patients, there was a significant rise in the average monocyte levels after each stage of RT, as compared to the preceding stage. However, the increase in lymphocyte, neutrophil, and eosinophil levels after each stage of RT was not statistically significant (Fig. 4).

**Effect of RT on** ***Rel-A*** **expression and white blood cells** 

The results of the mixed effects model show the relationship between the level of *Rel-A* expression and changes in the number of white blood cells (Table 4). The results also showed that increasing *Rel-A* expression level during the treatment period led to the increased level of eosinophils, neutrophils, lymphocytes, and monocytes by 0.000994, 0.419939, 0.199615, and 0.172627 fold, respectively. Remarkably, the changes of monocyte cells were statistically significant (p < 0.001; Table 4). The findings from the regression analysis showed that there was a statistically significant relationship between the expression of *Rel-A* and the quantity of monocytes and lymphocytes in the fourth week (p = 0.015; Table 5).

## DISCUSSION

Currently, over 50% of individuals diagnosed with cancer undergo radiation therapy at various stages of their treatment. Recent technological advancements have greatly improved the precision of dose delivery to the target tumor, making RT more effective in cancer treatment. On the other hand, tumor cells have been shown to develop radioresistance, which is linked to the increased recurrence and failure in therapy. While low-dose IR has been well defined for radioadaptive protection of normal cells, the exact mechanisms by which tumor cells develop adaptive resistance to the therapeutic fractional irradiation are unknown^[21]^. The use of radiation therapy in the treatment of BC has been a subject of prolonged debate and disagreement over the years^[^^22^^]^. To date, evidence has demonstrated that RT reduces local relapse, but reduction in relapse rates does not translate into a decrease in mortality. Many explanations have been proposed for this disparity, including the negative effects of RT on the immune system^[^^23^^]^. The initial evaluation of mortality risks through an analysis of individual patient data indicated that radiation therapy had minimal impact on mortality within the first 10 years of follow-up. However, there have been indications that RT may have adverse consequences over an extended period^[^^24^^]^.

*NF-κB* plays a crucial role in immune response, cell survival, and cancer development as a direct reaction to radiation exposure. It has been shown that *NF-κB* reduces the programmed cell death or apoptosis by promoting the expression of antiapoptotic proteins and antioxidant molecules linked to the increased radioresistance, whereas its deletion in mice results in hypersensitivity to IR-induced gastrointestinal damage^[^^9^^,^^10^^]^. *NF-κB* also regulates the production of a wide range of cytokines in a cell type-specific manner^[^^21^^]^. Various kinds of cytokines promote hematopoietic stem cell proliferation and survival, which ultimately increases bone marrow recovery and tissue regeneration after irradiation^[^^24^^]^. As a result, pharmacological activation of *NF-κB* may be considered as a radioprotection/mitigation strategy. At the clinical level, radiation-induced injuries were well described, and understanding the mechanism of tissue responses following radiation has received a lot of attention in the recent years. *NF-κB* has been currently recognized as a key player in several critical steps in the development of radiation countermeasures^[^^24^^,^^25^^]^. Hence, the use of RT in all cases cannot be definitively beneficial in the treatment of BC, and molecular markers would act as predictors of treatment response.

**Table 4:** Association of *Rel-A* expression and white blood cell count

**White blood cell**	**Estimate**	**Standard error**	**95% CI**	**T-value**	**p value**
Eosinophils	0.000994	0.012792	(-0.02; 0.03)	0.078	0.938
Neutrophils	0.419939	0.269338	(-0.12; 0.96)	1.559	0.124
Lymphocytes	0.199615	0.110000	(-0.02; 0.42)	1.815	0.075
Monocytes	0.172627	0.029006	(0.11; 0.23)	5.951	<0.001

**Table 5:** Association of *Rel-A* expression and white blood cell count during different weeks of treatment based on the regression analysis

**White blood cell**	**Time**	**Regression coefficient**	**Standard error**	**T-value**	**p value**
Eosinophils	Baseline	-0.293	0.408	-0.719	0.485
	1 Week	-0.244	0.164	-3.321	0.066
	2 Weeks	-0.067	0.120	-0.557	0.587
	4 Weeks	-0.177	0.090	-1.960	0.072
					
Neutrophils	Baseline	-11.112	9.967	-1.115	0.285
	1 Week	-3.054	4.331	-0.705	0.493
	2 Weeks	0.162	2.195	0.074	0.942
	4 Weeks	0.849	1.966	0.432	0.673
					
Lymphocytes	Baseline	1.389	4.704	0.295	0.772
	1 Week	-2.084	2.295	-0.908	0.380
	2 Weeks	1.583	0.759	2.085	0.057
	4 Weeks	1.507	0.538	2.800	0.015
					
Monocytes	Baseline	0.104	0.158	0.660	0.521
	1 Week	0.475	0.661	0.718	0.485
	2 Weeks	-0.038	0.214	-0.178	0.861
	4 Weeks	-1.896	0.733	-2.586	0.023

The main finding of this study was that the tumor samples had higher *Rel-A* and *PACER* expression level than the normal adjacent samples. We discovered that the expression of the *Rel-A* and *PACER* genes increased in each step of RT, while that of *miR-7* decreased. Given the tumor-promoting role of canonical *NF-κB* in cancer, the selective suppression of canonical *NF-κB* could be used in clinical therapy^[^^26^^,^^27^^]^. Existing screening methods cannot detect BC at the early stages. Therefore, initial detection and diagnosis are required for an effective treatment, and development of new biomarkers for early detection is critical. As the result, in the treatment strategy for BC, using the methods that can inhibit the *NF-κB* pathway seems essential. In the current study, we explored that RT can activate *NF-κB* pathway by decreasing the expression of *miR-7* and increasing the expression of *Rel-A* and *PACER* (Supplementary Fig. 1). Notably, activation of this pathway would trigger the recurrence of BC in patients. By interfering with immune tumor micro-environments, RT can also cause secondary cancers^[^^16^^]^. 

In certain types of cancer, increased *NF-κB* activity is linked to tumor resistance to chemotherapy and radiation^[^^28^^]^. Kufe and Weichselbaum^[^^29^^] ^have demonstrated for the first time that IR activates DNA binding of *NF-κB*. It has also been indicated that blocking the *NF-κB* activity increases apoptotic response and decreases the cell growth, as well as clonogenic viability, in several human cancer cell lines^[^^30^^,^^31^^]^. *NF-κB* suppression also enhances radiosensitivity^[^^32^^]^. NF-κB appears to affect the cell sensitivity to radio/chemotherapy in a cell type-specific manner^[^^32^^]^. Moreover, *NF-κB* is involved in adaptive radioresistance in mouse epidermal cells and human keratinocytes, and the inhibition of *NF-κB *activity prevents adaptive radioresistance^[^^32^^]^. Human BC cells exposed to fractioned irradiation have been shown to have increased clonogenic viability and NF-κB activation^[^^33^^]^. Together with the assumption that *NF-κB* can regulate more than 150 effector genes, it was hypothesized that *NF-κB* plays a key role in tumor radioadaptive resistance to fractional IR. This pro-survival network is initiated by *NF-κB* and related to the DNA-damage sensor protein *ATM*, the *EGFR* family epidermal growth factor receptor 2, and the mitochondrial antioxidant *manganese superoxide dismutase*^[^^34^^,^^35^^]^.

MiRNAs have the ability to control the components of human signaling pathways; however, much remains unknown about the function of miRNA in cancer^[^^36^^-^^40^^]^. Numerous studies have found that the expression of various miRNAs is altered in BC^[^^17^^-^^19^^]^. In different BC cell lin es, *miR-7* has been shown to suppress cell proliferation and migration and also induce apoptosis. Researches indicate that *miR-7* acts as a cancer suppressor, and when *miR-7* is artificially increased, it substantially diminishes the self-renewal capacity of BC stem cells^[^^17^^-^^19^^]^. In addition to being diagnostic markers, miRNAs can be used as markers for cancer treatment and radiation resistance. In 2011, researchers discovered that miRNAs can serve as biomarkers to predict treatment responses^[^^16^^]^. According to the findings of this study, *miR-7* had different expression levels before and after treatment, and these miRNAs targeted the key genes such as *EGFR*, *AKT*, and *Rel-A*, which all were involved in cancer radiation^[^^16^^]^. Due to the significant role that radiotherapy plays in treating cancer, there is considerable focus on investigating the mechanisms involved in tumor radioresistance. Growing evidence indicates that the presence of radioresistant cancer stem cells during the repopulation process may play a substantial role in the development of tumor radioresistance^[32]^. However, it appears that a Pro-survival pathway initiated by *NF-κB* is responsible for the radioadaptive response in the cells^[^^32^^,^^33^^]^. To fully realize the potential of *NF-κB* as a target for antitumor treatments, it is essential to acquire a through comprehension of the mechanisms that regulate particular *NF-κB* networks associated with adaptive radioresistance. Without this deeper knowledge, the promise of utilizing *NF-κB* as an effective strategy against tumors remains unfulfilled. At present, it is not clear if tumor repopulation and/or the selection of cancer stem cells by radiation therapy contribute to heterogenic *NF-κB* activation, which in turn is associated with tumor radioresistance. The exact relationship between these factors remains unknown^[^^16^^]^. The present study found a decreased *miR-7* expression level and increased expression levels of its target gene (*Rel-A*) following RT. Our study also showed that with increasing radiation dose, the expression of target genes increases over time. It should be noted that none of our patients had concurrent chemotherapy. Therefore, the altered expression of genes involved in the *NF-κB* pathway, including *Rel-A*/*PACER*/*miR-7*, could be used to predict the effects of therapeutic approaches.

**Fig. 5:**
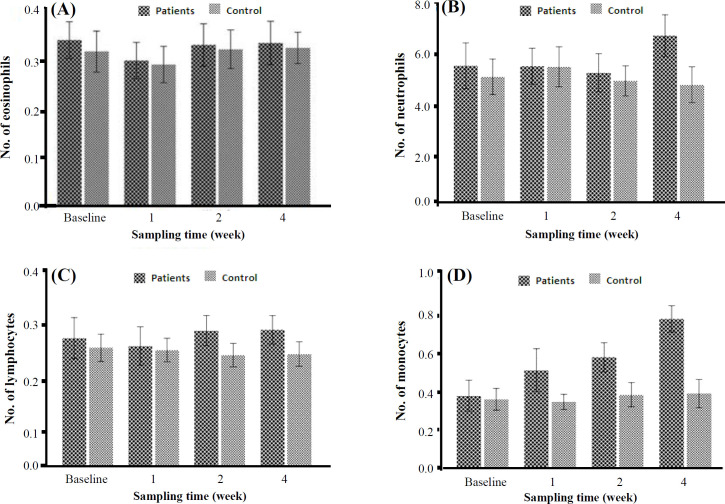
White blood cell and differential count for eosinophils (A), neutrophils (B), lymphocytes (C), and monocytes (D) in BC patients and control group. Data are representative of ± SD.

 In summary, our findings disclose that radiotherapy affects the *Rel-A/PACER/miR-7* axis in human BC, leading to the enhanced immunity-related signaling. This discovery suggests a promising new approach for countering the effects of ionizing radiation in types of cancer associated with this signaling network. Monitoring the expression level of the above-mentioned genes could potentially serve as a useful indicator to assess the efficacy of radiotherapy. However, further research with larger sample sizes is necessary to validate this hypothesis. 

## DECLARATIONS

### Acknowledgments

This research was supported by a grant (Grant number: IG-39711) from the Research Department of the Tarbiat Modares University, Tehran, Iran. The authors sincerely appreciate the head and staff of the Oncology Department of Imam Khomeini Hospital for their valuable cooperation. The authors also thank all patients and healthy volunteers who participated in this study.

### Ethical statement

The study protocol was approved by the Ethics Committee of the Tarbiat Modares University, Tehran, Iran (ethical code: IR.MODARES.REC.1400.232).

### Data availability

 The analyzed data sets generated during the study are available from the corresponding author on reasonable request.

### Author contributions

FS: conceived the idea and research strategy and involved in experiments and data collection and analysis; HM: conceived the idea and research strategy and supervised the research and verified data analysis; MDO: involved in data analysis and interpretation. All authors were involved in drafting, revision and final approval of the manuscript. 

### Conflict of interest

 None declared.

### Funding/support

There is no funding supported this project.

## Supplementary Materials


