# Supplementary material for: Rel-A/PACER/miR7 Axis May Play a Role in Radiotherapy Treatment in Breast Cancer Patients
Source: Iran Biomed J. 2023 May 8;27(4):173–82. doi: 10.61186/ibj.3901 (PMC10507291; doi:10.61186/ibj.3901)
Supplement: Supplementary file 1 [file ibj-27-173-s001.pdf]

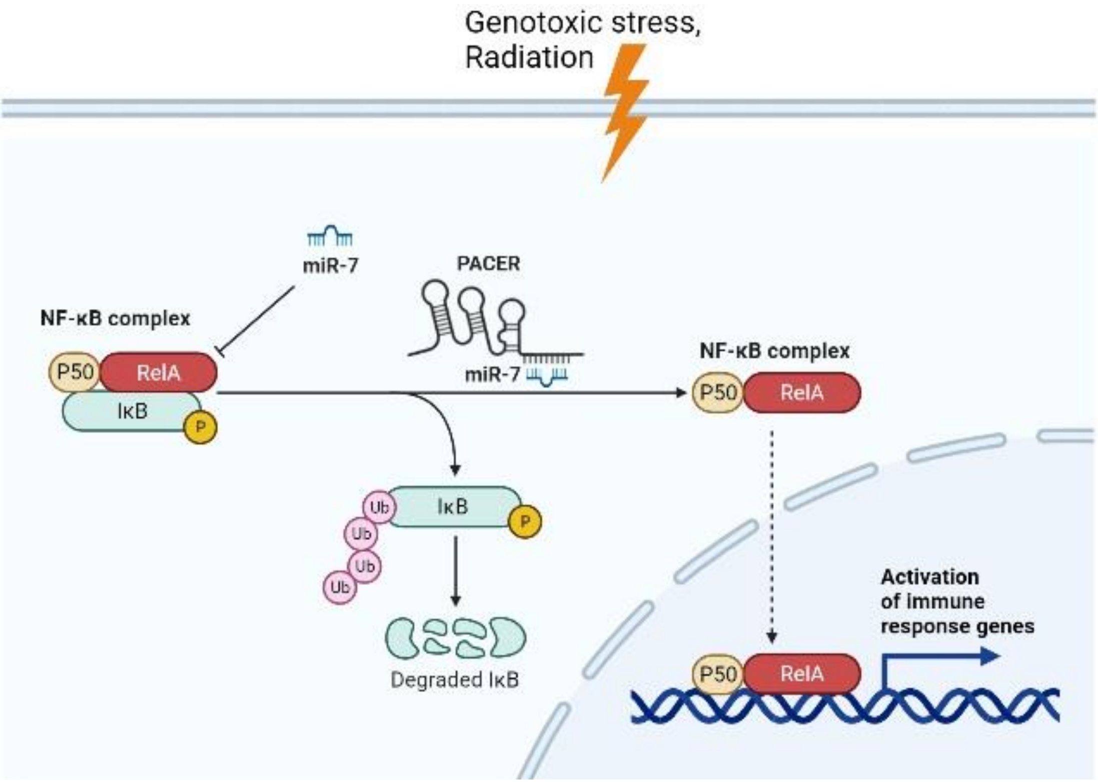

Supplementary Fig. 1. Schematic picture of nuclear factor (*NF-κB*) signaling pathway influencing the cellular responses to IR. *PACER* directly interacts with and sponges *miR-7* and inhibits *miR-7* to hybridize with *Rel-A*. Rel-A protein is transported to the nucleus, where it initiates the activation of genes responsible for the immune response.
